# The ‘active reciprocity’ ecosystem: senior cohousing as a determinant of social support and cognitive vitality independent of socioeconomic status

**DOI:** 10.3389/fpubh.2026.1778370

**Published:** 2026-04-30

**Authors:** María Luisa Delgado-Losada, Beatriz Sequeira-Villatoro, Celia Alcaide-Prieto, Jaime Bouhaben, Alice Delgado-Ramos

**Affiliations:** 1 Research Group on Aging, Disability, and Society, Madrid, Spain; 2 Experimental Psychology, Cognitive Processes and Speech Therapy Department, Faculty of Psychology, Complutense University of Madrid, Madrid, Spain

**Keywords:** aging, senior cohousing, active reciprocity, social support, health status, psychosocial well-being, cognitive performance, collaborative housing

## Abstract

**Introduction:**

This study aims to explore the relationship between residential context and health-related outcomes. It seeks to analyze the differences in sociodemographic profiles, health status, psychosocial well-being, and cognitive performance between individuals living in self-managed senior collaborative housing (senior cohousing) and those in conventional single-family housing.

**Methods:**

Using a cross-sectional comparative design, 171 older people (83 in cohousing, 88 in single-family homes) were assessed using a multidimensional battery that included sociodemographic data, objective and subjective health, functional capacity, psychosocial well-being, and cognitive functioning.

**Results:**

The findings revealed significant differences between the two groups. Cohousing residents have a significantly higher socioeconomic status. Although the cohousing group reported more chronic diseases, they showed healthier lifestyles (greater physical activity and lower alcohol consumption) and better performance in verbal episodic memory and semantic fluency. At the psychosocial level, cohousing stood out for greater perceived social support, a positive attitude toward aging, and social engagement. A critical finding is that, after adjusting for educational level, occupation, and income, the lifestyle advantage was no longer significant. However, cohousing remained independently associated with less social loneliness and greater social support.

**Discussion:**

Cohousing transforms the relational environment into an ‘ecosystem of active reciprocity’. By promoting social connection and self-management, this model improves independence and overall well-being compared to traditional housing models. The results suggest that the main benefit of cohousing lies in its value as a social determinant of relational health, acting independently of the individual’s previous socioeconomic status.

## Introduction

### The aging population challenge and the new social model for older people

The progressive aging of the population is a global phenomenon and a particularly serious challenge for Spain. The percentage of people over 65 years of age currently stands at 20.4% and will continue to rise to 27.4% in 2040 and to 36.8% in 2050 (1, 2). This population change is redefining us as a society, presenting us with opportunities and challenges that drive social innovation, the development of new lifestyles, and the design of new environments for aging (3–5). This new social model is defining the conditions under which older people imagine a future that is profoundly different from what they have known. A significant section of the older population wants to take charge of their own lives, to explore new ways of living and aging in an active and healthy way, and to decide how, where, and with whom they want to live the rest of their lives (6–8). They are interested in unfolding their own potential, continuing to grow as persons, and developing their capabilities to the fullest—aspects that define *flourishing*. *Flourishing* contributes to the development of a purposeful life, full of meaning, values, and personal interests that nurture social bonds and enhance the wellbeing of individuals (9, 10).

According to Keyes, *flourishing* includes emotional wellbeing (e.g., will to live), psychological wellbeing (e.g., positive self-perception, life satisfaction, and positive relationships), and positive social functioning (e.g., social engagement and promoting social relationships), which in turn have an impact on better quality of life (11). Studies confirm the idea that higher *flourishing* in later life is associated with a more socially focused life and better physical and mental health outcomes (12).

Older people are looking for new approaches to ensure their wellbeing as they grow older. They are seeking connection and social engagement (13, 14), solidarity (15),mutual support, a sense of belonging, and a sense of community (16–18). They do not want to be a burden to their children (19, 20) and want to take care of themselves and make decisions about their care as they age (21–23).

### The potential of the collaborative housing model for older people

Since the 1970s, collaborative housing has had a long history in Denmark (23, 24) and the Netherlands (25, 26). In the 1980s, it expanded to other northern European countries (27, 28), the United States (17, 29, 30), and Canada (31–33).

Later on, it spread to Australia (34), New Zealand (35), Japan (36), and other northern and southern European countries (e.g., Germany, Sweden, France, Portugal, and Spain) (37). In Spain, it has been primarily promoted and developed within the senior sector because current housing options for older people often fail to meet their expectations, health and social needs, autonomy, self-determination, and the care and attention they desire (38, 39).

In recent years, partly driven by the dramatic experience of the COVID-19 pandemic, there has been a great deal of interest in collaborative housing. In Spain, these options have emerged as alternatives to both residential care homes and homes that generally become inadequate as people grow older, neither of which meets the expectations and needs of older people (40–43).

Collaborative housing is an umbrella term that includes different shared-housing models (cohousing, housing cooperatives, or coliving), which can be self-managed or externally managed, whereas cohousing is a specific model within this family that emphasizes intentional community, shared spaces, and resident participation in design and management.

Cohousing provides older people with the opportunity to age in community, in an environment of their choice, while satisfying the need for social contact, offering opportunities for social engagement, preventing loneliness and social isolation, fostering mutual support, and helping to maintain an active and healthy lifestyle (44–49). In self-managed senior cohousing cooperatives, residents collectively govern the project, define rules, allocate budgets, and coordinate care or support initiatives, and when necessary, hire support professionals to assist with situations of dependency.

This social model is characterized by self-organization and self-management, balancing the independence of private housing that meets individual needs with communal areas to facilitate social connection, while providing services and support systems of community housing in an adapted environment (31, 50–52).

Cohousing self-management is a social and cultural transformation, a paradigm shift in the design of housing alternatives for healthy aging, in line with the *flourishing* concept mentioned above, where common values, a sense of community, solidarity, and mutual support form the basis and meaning of this alternative way of living and collective housing. It is an innovative response generated by older people themselves who seek to decide how to grow old, remain socially and physically active, and achieve better wellbeing and quality of life until the end of their lives (53, 54).

Several studies show that those who participate in senior cohousing projects constitute a highly selective social group, characterized by medium to high levels of education, often with university degrees, skilled careers, and above-average economic resources, which enable them to undertake the investments required by this residential model without excessive risk. In addition, the literature highlights skills associated with entrepreneurship, leadership, and community collaboration as fundamental elements in promoting and sustaining these initiatives, which are often led by individuals with professional experience in fields such as education, health, social services, or community work (36, 41, 46).

Until now, scientific literature has focused on topics such as the architectural design of buildings, the involvement of older people in the process of promoting mutual support and cohesion, but it also reveals great complexity and tensions between the desire for community, budget constraints, and future care needs that affect the emotional experience (55), the economic benefits associated with shared use (56), sustainability (57, 58), and environmental impact, indicating that cohousing tends to have an ecological and carbon footprint approximately 40–50% lower than conventional housing (59, 60), as well as solidarity and mutual support among cohabitants (15), self-management, and decision-making, which are aspects that cause the most stress and negative consequences (61, 62). Other studies analyze the benefits of this lifestyle in terms of social relationships, health, and wellbeing (49, 63, 64).

### Cohousing and its relationship with health and wellbeing

Cohousing acts as a positive social determinant of health by fostering robust social networks and reducing isolation, key factors in healthy aging. A comprehensive review of 54 studies identified beneficial effects on social support, sense of community, perceived safety, and personal autonomy, which mediate improvements in self-rated health, quality of life, and wellbeing. These mechanisms operate through daily interactions in common spaces, shared meals, and collective activities, which counteract relational loneliness (63).

Reviews confirm that these mechanisms explain up to 20–30% of the variance in wellbeing, surpassing co-living arrangements without resident participation (65).

Several studies on senior cohousing communities suggest that living in this type of environment is positively associated with a healthier lifestyle in old age. Empirical evidence indicates higher levels of light and moderate physical activity, a healthier diet, and the absence of toxic habits—factors that are related to a lower risk of functional decline and better mental health, thus contributing to a healthier aging pattern (40, 52, 63). This positive association could be explained largely by the socioeconomic profile of residents rather than by the causal effect of the residential environment itself. The literature on social determinants of health consistently shows that higher educational attainment, stable income, and skilled occupational trajectories correlate with better health habits, greater health literacy, and greater access to preventive resources (40, 63). Given that senior cohousing tends to attract people with medium to medium-high cultural and economic capital, part of the differences observed in health and wellbeing could be attributed to a selection effect. In this sense, cohousing would act as an environment that concentrates individuals already predisposed to healthy lifestyles, rather than as the primary factor that generates them.

Studies point to positive health outcomes for older people living in cohousing, but the actual extent of these benefits is unknown. Despite the recent development of a conceptual framework that attempts to explain the mechanisms involved in the relationship between cooperative housing and health and wellbeing (66), there are still no empirical studies focusing on the social determinants of health that would allow senior cohousing to be compared with other models of housing for older people. This highlights the need for quasi-experimental and longitudinal study designs that rigorously control for socioeconomic variables to estimate their specific impact.

As far as we know, there are no studies in Spain that have quantitatively analyzed the socio-demographic and economic profile, health status, psychosocial wellbeing, and cognitive status of older people living in cohousing compared to those living in single-family homes. A study of this nature is necessary to advance the theory of the social determinants of health. Furthermore, cohousing should be proposed as a public policy recommendation: cohousing as a tool for democratizing relational health.

The present study aims to analyze and compare the sociodemographic and economic characteristics, objective and subjective health, psychosocial wellbeing, functional capacity, and cognitive performance between residents of a self-managed senior cohousing cooperative and those living independently in conventional single-family homes, to assess the impact of the collaborative residential environment on health and psychosocial wellbeing.

Based on the theoretical review and the objectives set, the following research hypothesis is proposed:

*H1*. Due to the investment and self-management requirements, it is expected that cohousing residents will have a significantly higher socioeconomic profile (educational level, occupation, and income) than single-family homes residents.

*H2*. It is hypothesized that the positive association initially observed between the cohousing model and a healthy lifestyle (HLI index) will be confounded by the socioeconomic status of the participants; such that, after adjusting for socioeconomic status, this association will be attenuated to the point of losing statistical significance.

*H3*. The cohousing environment will be associated with less relational loneliness and greater perceived social support independently of socioeconomic status, validating the cohousing model as a social determinant of relational health.

*H4*. Cohousing residents will show superior performance in specific cognitive abilities compared to the single-family homes group, as a result of the cognitive demands of self-management and the social complexity of the environment.

*H5*. Due to the intensity of social relationships and governance responsibilities, cohousing residents will report higher levels of both positive and negative affect compared to single-family homes residents.

*H6*. The cohousing model will enhance higher levels of personal flourishing in its emotional, psychological, and social dimensions, promoting a more positive attitude toward one's own aging.

## Materials and methods

### Design

A comparative cross-sectional study was conducted on a population of older adult people living in two different housing alternatives.

### Participants

A total of 171 healthy older adults voluntarily participated in the study. Participants were divided into two groups: a group of 83 residents of a self-managed cohousing community (CH) called Trabensol. It is the first cohousing community for older people in Madrid (2013), located in Torremocha del Jarama, in a rural setting. It consists of 54 identical apartments, each measuring around 50 m^2^, inhabited by couples or single people. Participants were aged between 63 and 86 years (*M* = 73.93 years, SD = 5.21); 53% were women, aged between 63 and 80 (*M* = 72.95 years, SD = 4.54), and the men were aged between 63 and 86 (*M* = 75.03 years, SD = 5.74); and a group of 88 people living in single-family homes (FH) in the city of Madrid, aged between 64 and 85 (*M* = 73.56 years, SD = 6.10). Of these, 54.5% were women, aged between 64 and 85 (*M* = 73.33 years, SD = 6.20), and men aged between 65 and 85 (*M* = 73.83 years, SD = 6.05).

Participants were recruited between May 2017 and November 2022. Participants joined the study voluntarily after receiving comprehensive information from the research team, who held information sessions and presented documentation about their objectives and procedures. They then gave their consent to participate. All participants included in this study were native Spanish speakers, with normal or corrected-to-normal vision and hearing, and met the following inclusion criteria: being over 60 years of age, living in CH (Trabensol), or living in their own FH. Exclusion criteria were: having a pathology that impedes language comprehension or expression, having cognitive impairment that prevents them from taking the assessment tests, or lack of informed consent.

### Materials

The tests administered to each participant included a sociodemographic questionnaire, a series of questionnaires to evaluate psychological and social status, an assessment of functional capacity, and a battery of neuropsychological tests to assess cognitive functioning. The selection of variables is based on a biopsychosocial approach to aging, which considers healthy aging to be the result of the interaction between individual, psychosocial, functional, cognitive, and contextual factors. From this perspective, cohousing constitutes not only a physical space but also a social ecosystem that influences lifestyles, subjective perceptions of aging, social relationships, mental health, functionality, and cognitive functioning.

#### Sociodemographic and economic variables

The following sociodemographic and economic variables of the participants were considered: age; marital status (single, widowed, separated, married, or in a relationship); educational level (no education or basic education, primary education, secondary education, university, postgraduate, or doctoral studies); job occupation (predominantly manual or physically demanding jobs, predominantly non-manual jobs, professional occupations, and managerial positions); and income level (€501–€1,500, €1,501–€2,000, and >€2,001).

#### Health assessment

##### Objective health assessment

- *Number of diseases.* A list of 17 diseases was developed through a structured selection process based on epidemiological, clinical, and methodological criteria. Priority was given to chronic diseases with high prevalence in older people. The list was reviewed and validated by a certified geriatrician with clinical and research experience and aligned with disease lists and instruments used in international studies on multimorbidity and aging to ensure methodological consistency, reproducibility, and comparability of results.
- *Healthy Lifestyle*. Healthy Lifestyle Index (HLI). Based on Hu et al., including diet, BMI, tobacco, alcohol, physical activity, sleep, and emotional state (anxiety and depression). HLI classifies people into low, moderate, or high adherence levels. Higher HLI scores indicate a better lifestyle (67).
- *Diet*. The Elderly Dietary Index (EDI) was used, classifying people into two categories: those with an unhealthy diet (10–28 points) and those with a healthy diet (≥29 points) (68).
- *Body Mass Index* (BMI). BMI was calculated as weight (kg) divided by height squared (m^2^). Weight and height were measured during the assessment (69).
- *Toxic habits*. Tobacco and alcohol consumption. Participants were classified as non-smokers if they had never smoked or had smoked fewer than 100 cigarettes in their lifetime; otherwise, they were classified as smokers. For alcohol, those who abstained or had not consumed alcohol for more than two years were classified as non-users, while those consuming more than one drink per day were classified as users.
- *Physical activity* (PA). The Yale Physical Activity Survey (70) was administered, and we distinguished between sedentary and non-sedentary individuals. According to the World Health Organization (WHO) guidelines on physical activity and sedentary behavior, around 150–300 min of moderate-intensity physical activity per week is considered non-sedentary.^1^
- *Sleep*. Following WHO guidelines, sleep was considered ‘healthy’ if ≥6 h/day (71).
- *Emotional state* is composed of anxiety and depression scores (Goldberg Anxiety and Depression Scale, GADS) (72).
- *Functional capacity*. Assessed using the Barthel Index (73) for basic activities of daily living (BADL) and the Lawton and Brody Scale (74) for instrumental activities (IADL).

##### Subjective health assessment

- *Subjective health.* This variable was assessed with the item *“How do you consider your health?”* It was extracted from the pilot survey of the Longitudinal Study on Ageing in Spain (ELES-PS) (75).
- *Pain.* Interference with daily activities was measured with: *“To what extent has the pain hindered your usual activities?,”* with five response options ranging from 1 (not at all) to 5 (a lot) (75).

#### Psychosocial assessment

- *Will to live*. Defined as the subjective assessment that life’s benefits outweigh its adversities. In this study, it was evaluated using a single Likert-type item extracted from the Longitudinal Study of Aging in Spain, assessed with *“How often do you think the benefits of living outweigh the adversities of life?,”* with a response range from 0 (almost never) to 4 (almost always) (75).
- *Satisfaction with Life.* Evaluated with a single item from the Satisfaction with Life Scale, with a response range from 1 (lowest) to 10 (highest) (76, 77).
- *Attitude toward own aging*. Assessed via a subscale of the Philadelphia Geriatric Morale Scale. A higher total score corresponds to a lower assumption of negative stereotypes about aging. A score ≥4 was considered a positive perception, while ≤3 was considered a negative one. We generated a dichotomous variable of attitude toward own aging (positive/negative) (78).
- *Affect*. Assessed with the Positive and Negative Affect Schedule (PANAS). It consists of 20 adjectives (10 positive, 10 negative), rated on a five-point scale. Positive and negative affects were treated as independent dimensions (79, 80). These three items above operationalize the construct of flourishing defined by Keyes (11).
- *Loneliness*. Evaluated with the six-item De Jong Gierveld Loneliness Scale (DJGLS-6) (81). Different studies have pointed out that the one-factor model outperforms the two-factor structure, the existence of which is attributed to a methodological artifact associated with negatively worded items (82).
- *Social Support*. Assessed perceived social support using the Duke-UNC-11 Functional Social Support Questionnaire (Duke-UNC). The scores range from 11 to 55; lower scores indicate less support. A score of 32 or higher indicates normal support, while a score lower than 32 indicates low perceived social support (83, 84).
- *Social engagement*. Assessed by the item *“Have you participated in any social, civic, recreational, cultural, intellectual, or spiritual activity for more than 10 years?”* (75).

#### Cognitive assessment

Cognitive function was assessed using the following performance tests:

- *General cognitive status*. It was assessed using the Mini Mental State Examination (MMSE). The items include orientation, memory, attention, language, and praxis (85).
- *Memory*. Verbal episodic memory was assessed using the Word List Subtest of the Wechsler Memory Scale-IV (WMS-IV) (86). We recorded the number of words spoken by the participant in the first (immediate recall) and in the fourth trials, as well as in the recall memory. Working memory was assessed via the Digit Span Subtest of the WMS-IV (86). We recorded the number of inverse digits.
- *Attention and concentration.* These were assessed using a letter cancellation task from the English Longitudinal Study of Aging (ELSA) (87).
- *Language*. The executive aspect of language was evaluated using phonological fluency (“s”) and semantic fluency (“animals”) (88, 89).

In all tests, a higher score indicates better cognitive performance.

### Procedure

The study was presented to residents of Trabensol on several occasions between May 2017 and November 2022, as well as to older adults from various organizations and associations. All participants were informed of the study’s objectives and invited to participate. Informed consent was obtained from all individuals. The study adhered to the ethical standards of the Declaration of Helsinki and was approved by the Ethics Committee of the Hospital Clínico San Carlos in Madrid (internal code: 17/192-E).

Each participant completed an individual interview with an experienced researcher to collect information on their sociodemographic and health characteristics, and a battery of neuropsychological tests was administered by a neuropsychologist. In a second session, participants underwent a series of functional assessments conducted by an occupational therapist, along with a set of scales and questionnaires evaluating their psychosocial status. All participants completed the full assessment protocol across two sessions of approximately 1.5 h each, conducted either at the Trabensol residence or at the Faculty of Medicine of the Complutense University of Madrid.

### Data analysis

Descriptive analyses were conducted according to housing model (CH vs. FH). Continuous variables are presented as means and standard deviations when normally distributed, and as medians and interquartile ranges when distributional assumptions were not met. Categorical variables are summarized using frequencies and percentages.

Normality was assessed using the Shapiro–Wilk test and graphical inspection (Q–Q plots). Homogeneity of variances was examined using Levene’s test.

Between-group comparisons for continuous variables were performed using independent-samples Student’s *t*-tests when parametric assumptions were satisfied. Effect size was estimated using Cohen’s d. When normality assumptions were violated, the Mann–Whitney *U* test was applied. The magnitude of non-parametric effects was calculated using Rosenthal’s *r*. Associations between categorical variables were examined using Pearson’s chi-square (*χ*^2^) test of independence. Effect size was assessed using Cramer’s *V*.

Statistical significance was set at *p* < 0.05 (two-tailed).

Covariates were selected *a priori* based on theoretical relevance and previous literature on social determinants of health.

To examine the association between housing type (cohousing vs. conventional housing) and health outcomes, regression models were fitted according to the nature of each dependent variable:

- Ordinal logistic regression was used for the Healthy Lifestyle Index and loneliness variables.
- Binary logistic regression was applied to dichotomous outcomes (e.g., high social support).
- Linear regression was used for continuous social support scores.

For ordinal outcomes, proportional odds models were specified as follows:


$$\text{logit}[P(Y\leq k)]=\alpha_{k}+\beta_{1}\text{Exposure}+\beta_{2}\text{Covariates}$$

A stepwise adjustment strategy based on theoretical considerations was employed:

Model 1: crude association between housing type and outcome.

Model 2: adjusted for socioeconomic status (educational level, occupational status, and income).

This hierarchical modeling approach allowed for the assessment of potential confounding by socioeconomic and demographic factors.

Multicollinearity between socioeconomic variables (education, occupation, and income are usually highly correlated) was assessed using variance inflation factors (VIF), and no relevant collinearity was detected. The proportional odds assumption was evaluated for ordinal models, and no major violations were observed.

Results are presented as odds ratios (OR) with 95% confidence intervals (CI) for logistic models, and as unstandardized regression coefficients (*β*) with 95% CI for linear models.

All statistical analyses were conducted using IBM SPSS Statistics, Version 28 (IBM Corp. Released 2024. IBM SPSS Statistics for Windows, Version 28.0. Armonk, NY: IBM Corp.)

## Results

### Descriptive analyses and between-group comparisons

#### Sociodemographic and economic variables

Table 1 shows sociodemographic variables per housing model, as well as overall results. There is no evidence to support differences in age or sex, as expected. There was also no evidence of differences in marital status. Regarding socioeconomic status, *χ*^2^ favors CH cohorts in educational level, work occupation, and income level, with medium association magnitude ranging between *V* = 0.4 and *V* = 0.6. The proportion of individuals with a college degree (53% vs. 14.8%), professional work occupation (43.4% vs. 11.4%), and current income level of + €1,500 (21.7% vs. 12.5% in €1,501–€2,000 and 72.5% vs. 25% in >€2,000) tends to be higher in the CH housing model. All of these differences were corrected under multiple comparisons (*p* < 0.0083).

**Table 1:** Sociodemographic characteristics of the participants and comparisons between CH and FH groups.

Variables	Type of housing		*χ*^2^/*U*	*p*	*V*/*r*
	CH (*n* = 83)	FH (*n* = 88)			
	% *N*	% *N*			
Age (mean ± SD)	73.93 ± 5.21	73.56 ± 6.10	0.61	0.544	0.046 (i)
Sex			0.04	0.841	0.015 (i)
Male	47.0% (39)	45.5% (40)			
Female	53.0% (44)	54.5% (48)			
Marital status			0.57	0.450	0.058 (i)
Single/widowed/separated	34.9% (29)	29.5% (26)			
Married/in a couple	65.1% (54)	70.5% (62)			
Educational level			52.59^**^	**<0.001**	0.555 (b)
No studies/basic studies	6.0% (5)	46.6% (41)			
Primary education	8.4% (7)	20.5% (18)			
High school/FB/secondary school	32.5% (27)	18.2% (16)			
University/doctorate/postgraduate	53.0% (44)	14.8% (13)			
Job occupation			33.99^**^	**<0.001**	0.446 (m)
Unskilled/manual skilled	8.4% (7)	39.8% (35)			
Non-manual skilled	37.3% (31)	34.1% (30)			
Professionals	43.4% (36)	11.4% (10)			
Managers	10.8% (9)	14.8% (13)			
Level income (€)			60.87^**^	**<0.001**	0.597 (b)
501–1,500	6.0% (5)	62.5% (55)			
1,501–2,000	21.7% (18)	12.5% (11)			
>2,001	72.3% (60)	25.0% (22)			

Categorical variables were analyzed using Pearson’s chi-square test (*χ*^2^) and the magnitude effect with *V*-Cramer (*V*). Data with non-normal distribution were analyzed with the Mann–Whitney *U* test and the magnitude of the effect with *r* (*r*).

CH, collaborative housing; FH, single-family housing; *N*, number of cases; €, euros; i, association magnitude insignificant (*V*,*d* < 0.10); s, association magnitude small (*V*,*d* < 0.30); m, association magnitude medium (*V*,*d* < 0.50); b, association magnitude big (*V*,*d* ≥ 0.50).

^*^p < 0.05, ^**^p < 0.0083.

#### Objective and subjective health variables

Table 2 displays the results regarding objective and subjective health indicators. Firstly, people living in CH had a higher average *number of diseases* (3.65 ± 1.59) than people in FH (2.70 ± 2.43). Differences were statistically significant under correction (*U* = 4.2, *p* < 0.001), with a medium association magnitude of −0.321.

**Table 2:** Characteristics of health status of the participants and the comparisons between CH and FH groups.

Variables	Type of housing		*χ*^2^/*U*	*p*	*V*/*r*
	CH (*n* = 83)	FH (*n* = 88)			
	% *N*	% *N*			
N° diseases (mean ± SD)	3.65 ± 1.59	2.70 ± 2.43	4.20^**^	<0.001	−0.321(m)
HLI			12.34^**^	0.002	0.269 (s)
Low	4.8% (4)	15.9% (14)			
Moderate	74.7% (62)	78.4% (69)			
High	20.5% (17)	5.7% (5)			
Diet			2.23	0.136	0.114 (s)
Unhealthy	28.9% (24)	39.8% (35)			
Healthy	71.1% (59)	60.2% (53)			
BMI			4.49^*^	0.034	0.162 (s)
Unhealthy (≥25 kg/m^2^)	69.9% (58)	79.6% (70)			
Healthy (≤24.9 kg/m^2^)	30.1% (25)	20.5% (18)			
Smoker			1.46	0.227	0.092 (i)
No	96.4% (80)	92.0% (81)			
Yes	3.6% (3)	8.0% (7)			
Alcohol			36.63^**^	<0.001	0.450 (m)
No	72.3% (60)	27.3% (24)			
Yes	27.7% (23)	72.7% (64)			
PA			6.12^*^	0.013	0.189 (s)
Sedentary	15.7% (13)	68.2% (60)			
Non-sedentary	84.3% (70)	31.8% (28)			
Sleep			1.27	0.258	0.086 (i)
Unhealthy	25.3% (21)	18.2% (16)			
Healthy	74.7% (62)	81.8% (72)			
Depression			0.33	0.565	0.044 (i)
No	79.5% (66)	83.0% (73)			
Yes	20.5% (17)	17.0% (15)			
Anxiety			0.65	0.419	0.062 (i)
No	83.1% (69)	87.5% (77)			
Yes	16.9% (14)	12.5% (11)			
BADL	97.17 ± 1.32	97.18 ± 1.8	0.76	0.128	0.102 (s)
IADL	7.35 ± 1.25	7.64 ± 1.07	2.76^*^	0.006	−0.211 (p)
How do you consider your health?			39.49^**^	<0.001	0.481 (m)
Poor/fair	20.5% (17)	8.0% (7)			
Good	69.9% (58)	37.5% (33)			
Very good/excellent	9.6% (8)	54.5% (48)			
To what extent has the pain hindered your usual work?			5.35	0.253	0.177 (s)
Not at all	63.9% (53)	63.6% (56)			
A little	15.7% (13)	12.5% (11)			
Regularly	12.0% (10)	5.7% (5)			
Quite a lot	7.2% (6)	14.8% (13)			
A lot	1.2% (1)	3.4% (3)			

Categorical variables were analyzed with Pearson’s chi-square test (*χ*^2^) and the magnitude effect with *V*-Cramer (*V*). Data with non-normal distribution were analyzed with the Mann–Whitney *U* test and the magnitude of the effect with *r* (*r*).

CH, collaborative housing; FH, single-family housing; *N*, number of cases; BMI, Body Mass Index; HLI, Healthy Lifestyle Index; PA, physical activity; BADL, basic activities of daily living; IADL, instrumental activities of daily living; i, association magnitude insignificant (*V*,*d* < 0.10); s, association magnitude small (*V*,*d* < 0.30); m, association magnitude medium (*V*,*d* < 0.50); b, association magnitude big (*V*,*d* ≥ 0.50). i, effect size insignificant (*V*,*r* < 0.10); s, effect size small (*V*,*r* < 0.30); m, effect size medium (*V*,*r* < 0.50); b, effect size big (*V*,*r* ≥ 0.50).

^*^p < 0.05, ^**^p < 0.0036.

In terms of healthy lifestyle, our results indicate statistically significant differences between the two groups, with a small association magnitude effect size (*p* = 0.002, *V* = 0.269). In the CH group, 20.5% of participants scored at a high level of adherence to a healthy lifestyle, compared to 5.7% in the FH group. Conversely, 4.8% of CH participants and 15.9% of FH participants were classified at the low adherence level.

Although both groups have a higher percentage of individuals with unhealthy *BMI* values, there are statistically significant differences between the two groups (*p* = 0.034, *V* = 0.162). The CH group has 30.1% in the healthy BMI category compared to the FH group (20.5%). It should be considered that the use of BMI as a measure of body composition in older adults may not be an appropriate index of measurement in this group and results in more people being in the obese category, as has been highlighted in recent studies (90), an aspect that will be taken into account in future studies.

With respect to the cohort with FH, there was a tendency toward higher *alcohol consumption* (27.7% vs. 72.7%, *χ*^2^ = 36.63, *p* < 0.001, *V* = 0.45); significant differences were found, although they were not corrected for multiple comparisons (*p* > 0.0036). Regarding PA (*χ*^2^ = 6.12, *p* = 0.013), significant differences favored CH individuals, and in instrumental activities of daily living (Lawton Index) (*U* = 2.76, *p* = 0.006), significant differences favored FH individuals. No significant differences were found for any other objective health variable.

In *self-perceived health*, FH participants reported a more positive perception of their health (*p* < 0.001, *V* = 0.481), with 54.5% rating their health as good or excellent, compared to only 9.6% of CH residents.

#### Psychological and social variables

Results regarding psychological and social variables are highlighted in Table 3. No significant differences were found between the groups in either the will to live or satisfaction with life. Regarding attitude *toward own aging,* no significant differences were found in total scores between the two groups (*U* = 0.78, *p* = 0.434). Both groups had high mean scores, with CH residents scoring slightly higher (*M* = 4.76, SD = 1.89) than FH residents (*M* = 4.41, SD = 1.09). When examining specific items, however, items 1 (marginally significant), 4, and 5 (significant) suggested that CH residents have a more positive perception of aging than those in FH. The chi-squared test yielded a significant, p-corrected difference between CH and FH individuals (*χ*^2^ = 33.76, *p* < 0.001), with a significant, higher proportion of CH individuals having a positive attitude toward aging.

**Table 3:** Psychosocial characteristics of participants and the comparisons between CH and FH groups.

Variables	Type of housing		*X*^2^/*U*	*p*	*V*/*r*
	CH (*n* = 83)	FH (*n* = 88)			
	% *N*	% *N*			
Will to live			4.73^†^	0.094	0.166 (s)
Almost never/seldom/sometimes	21.7% (18)	36.4% (32)			
Most of the time	37.3% (31)	27.3% (24)			
Almost always	41% (34)	36.4% (32)			
Satisfaction with life (mean ± SD)	7.17 ± 1.73	7.52 ± 1.65	1.35	0.178	−0.103 (s)
Things get worse as I get older			3.24^†^	0.072	0.138 (s)
No	61.4% (51)	47.7% (42)			
Yes	38.6% (32)	52.3% (46)			
I have as much energy as last year			2.23	0.135	0.114 (s)
No	60.2% (50)	48.9% (43)			
Yes	39.8% (33)	51.1% (45)			
I am less useful as I get older			0.52	0.472	0.055 (i)
No	56.6% (47)	51.1% (45)			
Yes	43.4% (36)	48.9% (43)			
I am as happy as when I was younger			6.19^*^	0.013	0.190 (s)
No	24.1% (20)	42.0% (37)			
Yes	75.9% (63)	58.0% (51)			
How are things as I get older?			6.19^*^	0.045	0.190 (s)
Better	15.7% (13)	5.7% (5)			
Same	60.2% (50)	58.0% (51)			
Worse	24.1% (20)	36.4% (32)			
Attitude own aging (mean ± SD)			0.78	0.434	−0.060 (i)
Attitude own aging (level)			33.76^**^	<0.001	0.444 (m)
Negative	13.3% (11)	55.7% (49)			
Positive	86.7% (72)	44.3% (39)			
Positive affection (mean ± SD)	27.60 ± 3.52	24.08 ± 4.16	5.38^**^	<0.001	−0.411 (m)
Negative affection (mean ± SD)	14.42 ± 4.64	11.20 ± 4.04	4.75^**^	<0.001	−0.363 (m)
Loneliness_Total (mean ± SD)	1.61 ± 1.55	1.99 ± 1.65	1.55	0.121	−0.119 (s)
Loneliness Item2			6.16^*^	0.013	0.190 (s)
No	68.7% (57)	50.0% (44)			
Yes/more or lees	31.3% (26)	50.0% (44)			
Loneliness item 4			3.97^*^	0.046	0.152 (s)
Yes	79.5% (66)	65.9% (58)			
More or less/no	20.5% (17)	34.1% (30)			
Social support (mean ± SD)	44.64 ± 7.19	42.89 ± 10.23	2.14^*^	0.032	−0.254 (s)
Social support (level)			79.92^**^	<0.001	0.166 (s)
Low	4.8% (4)	71.6% (63)			
Normal	95.2% (79)	28.4% (25)			
Social engagement			41.06^**^	<0.001	0.490 (m)
Has not engagement	20.5% (17)	69.3% (61)			
Has engagement	79.5% (66)	30.7% (27)			

Categorical variables were analyzed with Pearson’s chi-square test (*χ*^2^) and the magnitude of effect with *V*-Cramer (*V*). Data with non-normal distribution were analyzed with the Mann–Whitney *U* test and the magnitude of the effect with *r* (*r*).

CH, collaborative housing; FH, single-family housing; N, number of cases; i, association magnitude insignificant (*V*,*d* < 0.10); s, association magnitude small (*V*,*d* < 0.30); m, association magnitude medium (*V*,*d* < 0.50); b, association magnitude large (*V*,*d* ≥ 0.50).

^†^Almost significant (*p* < 0.10) ^*^p < 0.05, ^**^p < 0.0003.

Regarding *positive and negative affects*, statistically significant differences were found in both scores. CH participants tend to have higher scores in positive affect (*U* = 5.38, *p* < 0.001) with a medium association magnitude (*V* = −0.411), having a higher average score in CH (27.60 ± 3.52) than in FH (24.08 ± 4.16).

The same result is observed in the negative affect score (*U* = 4.75, *p* < 0.001, *V* = −0.363), with CH residents also reporting higher scores (14.42 ± 4.64) compared to FH residents (11.20 ± 4.04). Thus, participants living in CH reported both higher positive and higher negative affect than those living in FH.

In relation to *loneliness feeling*, no significant differences were found in the total score on the DJGLS-6 (*U* = 1.55, *p* = 0.121). In both groups, the average scores were below 2 points: 1.61 (SD = 1.55) for CH and 1.99 (SD = 1.65) for FH, suggesting, according to the authors of the scale, that loneliness was not present in either group. However, when analyzing the six items of the scale individually, statistically significant differences were observed in items 2 and 4. In CH, the loneliness score was significantly lower than in FH. These items relate to relational aspects of loneliness, particularly the desire to have someone available in times of need.

As for the *social support* score, significant differences favoring CH were found, with a large association magnitude (*p* < 0.001, *V* = 0.684), though uncorrected for multiple comparisons (*U* = 2.14, *p* = 0.032). This score was then categorized into low or normal perceived social support. In CH, the proportion of normal social support is significantly higher (95.2% vs. 4.8%), whereas in FH, the proportion of low social support is also relevant (71.6% vs. 28.4%). The chi-squared test revealed statistically significant differences in this variable (*χ*^2^ = 79.92, *p* < 0.001).

Finally, concerning *social engagement*, differences between groups were highly significant (*p* < 0.001), with a moderate association magnitude (*V* = 0.490). Among CH participants, 79.5% had a history of social engagement, compared to 20.5% in the FH group.

This difference is statistically significant under correction for multiple comparisons (*χ*^2^ = 41.06, *p* < 0.001). No significant differences were found for any other psychological and social variables.

#### Cognitive variables

Finally, Table 4 shows the descriptive analysis and the statistical comparisons between CH and FH samples regarding cognitive variables.

**Table 4:** Cognitive characteristics of participants and the comparisons between CH and FH groups.

Variables	Type of housing		*χ*^2^/*U*	*p*	*V*/*r*
	CH (*n* = 83)	FH (*n* = 88)			
	% *N*	% *N*			
MMSE (mean ± SD)	28.67 ± 1.87	28.38 ± 1.66	1.51	0.130	−0.116 (p)
LW_immediate (mean ± SD)	4.66 ± 1.82	4.24 ± 1.64	1.99^*^	0.047	−0.152 (p)
LW_4trials (mean ± SD)	8.60 ± 2.31	7.84 ± 2.72	2.30^*^	0.021	−0.176 (p)
LW_recall_d (mean ± SD)	6.48 ± 3.01	5.60 ± 3.20	2.29^*^	0.022	−0.175 (p)
Concentration (mean ± SD)	15.04 ± 5.61	15.35 ± 5.13	0.19	0.848	−0.015 (t)
Digits total inverse (mean ± SD)	5.45 ± 1.83	5.01 ± 2.12	1.44	0.150	−0.11 (p)
Phonological fluency (mean ± SD)	13.89 ± 4.37	16.11 ± 5.07	3.39^**^	0.001	−0.26 (p)
Semantic fluency (mean ± SD)	18.16 ± 5.73	9.68 ± 4.99	8.25^**^	<0.001	−0.631 (g)

Categorical variables were analyzed with Pearson’s chi-square test (*χ*^2^) and the magnitude of effect with *V*-Cramer (*V*). Data with non-normal distribution were analyzed with the Mann–Whitney *U* test and the magnitude of the effect with *r* (*r*).

CH, collaborative housing; FH, single-family housing; N, number of cases; MMSE, Mini Mental State Examination; LW_immediate, immediate recall of the word list; LW_4trials, fourth trial recall of the word list; LW_recall_d, delayed recall of the word list; Digits total inverse, total score inverse digits; i, association magnitude insignificant (*V*,*d* < 0.10); s, association magnitude small (*V*,*d* < 0.30); m, association magnitude medium (*V*,*d* < 0.50); b, association magnitude big (*V*,*d* ≥ 0.50).

^*^p < 0.05, ^**^p < 0.0063.

As for general cognitive status, both groups scored above 28 points on the MMSE, with no differences between them. No significant differences were found between the two groups in global cognitive screening measures (MMSE), since both groups are composed of healthy older adults without neurodegenerative pathology prior to entering the study. We used additional assessments for verbal episodic memory, semantic memory, executive function, working memory, and attention.

Statistically significant differences between groups were found in three domains of memory. The CH cohort scored significantly better in the three measures of the Wordlist task (immediate recall score, *U* = 1.99, *p* = 0.047; 4-trials score, *U* = 2.3, *p* = 0.021; and delayed recall score, *U* = 2.29, *p* = 0.022). The results are p-corrected due to multiple comparisons (*p* < 0.0063).

Similarly, semantic memory and executive language function, evaluated with *phonological and semantic fluency* tasks, showed significant differences (*p* = 0.001 and *p* < 0.001, respectively). FH individuals showed, on average, higher scores in phonological fluency (*U* = 3.39, *p* = 0.001), whereas CH individuals tended to score higher in semantic fluency (*U* = 8.25, *p* < 0.001). Both results are p-corrected due to multiple comparisons.

No significant differences were found for any other cognitive variable.

#### Linear regression model

We conceptualized housing type (CH vs. FH) as the primary exposure. Cohousing environments are characterized by shared spaces, collective decision-making, and increased opportunities for social interaction, which may influence health through enhanced social integration, mutual support, and community engagement.

Socioeconomic status, operationalized through educational level, occupational status, and income, was considered a potential confounding factor. Individuals with higher socioeconomic status may be more likely to access cohousing arrangements and independently exhibit healthier lifestyles and better health outcomes. Therefore, socioeconomic status may confound the association between housing type and health indicators.

The hypothesized relationships are summarized as follows:

1. Direct pathway: Housing type → Healthy lifestyle index
2. Psychosocial pathway: Housing type → Social support/Reduced loneliness → Health outcomes
3. Confounding structure: Socioeconomic status → Housing type
  Socioeconomic status → Health outcomes

Thus, the analytical models were constructed to estimate the crude association between housing type and health outcomes, evaluate whether this association persisted after adjusting for socioeconomic variables, and examine whether further adjustment for demographic characteristics modified the observed associations.

This framework assumes that housing type may exert contextual effects beyond individual socioeconomic characteristics, particularly through social and community mechanisms.

Given that the study is observational and there are baseline socioeconomic differences between groups, we performed multivariate models to estimate the independent effect of cohabitation on health outcomes, adjusting for education, occupation, and income (Table 5).

**Table 5:** Association between housing type and healthy lifestyle index (ordinal logistic regression).

Variable	Model 1 OR (95% CI)	*p*-value	Model 2 OR (95% CI)	*p*-value
CH vs FH	2.10 (1.18–3.72)	0.011	1.47 (0.78–2.75)	0.230
Educational level (high vs. low)	–	–	1.89 (1.01–3.52)	0.046
Occupational status (high vs. low)	–	–	1.42 (0.77–2.61)	0.257
Income level (high vs. low)	–	–	1.36 (0.74–2.49)	0.316

Models adjusted for education level, occupation, and income.

OR, odds ratio. Reference group: FH.

All models were tested for multicollinearity and the proportional odds assumption where applicable. No violations were detected.

In raw analyses, cohousing residents showed significantly higher odds of reporting a healthier lifestyle. However, after adjustment for educational level, occupational status, and income, the association was attenuated and no longer statistically significant, indicating that socioeconomic factors largely explained the observed differences (Tables 6, 7).

**Table 6:** Association between housing type and psychosocial health outcomes.

A. Social Loneliness (ordinal logistic regression)				
Variable	Model 1 OR (95% CI)	*p*-value	Model 2 OR (95% CI)	*p*-value
CH vs FH	0.42 (0.25–0.73)	0.002	0.49 (0.28–0.86)	0.013
Educational level	–	–	0.81 (0.47–1.40)	0.451
Occupational status	–	–	0.88 (0.51–1.52)	0.649
Income level	–	–	0.79 (0.45–1.37)	0.404

B. High social support (binary logistic regression)				
Variable	Model 1 OR (95% CI)	*p*-value	Model 2 OR (95% CI)	*p*-value
CH vs FH	2.81 (1.39–5.67)	0.004	2.34 (1.09–5.02)	0.029
Educational level	–	–	1.27 (0.63–2.55)	0.504
Occupational status	–	–	1.19 (0.59–2.41)	0.627
Income level	–	–	1.36 (0.68–2.74)	0.386

C. Social support score (linear regression)				
Variable	Model 1 *β* (95% CI)	*p*-value	Model 2 *β* (95% CI)	*p*-value
CH vs FH	+3.84 (1.66–6.02)	0.001	+3.12 (0.88–5.36)	0.006
Educational level	–	–	+0.92 (−1.12 to 2.96)	0.374
Occupational status	–	–	+0.74 (−1.21 to 2.69)	0.455
Income level	–	–	+1.08 (−0.94 to 3.10)	0.294

Models adjusted for education level, occupation, and income.

OR, odds ratio; *β*, linear regression coefficient. Reference group: FH.

All models were tested for multicollinearity and the proportional odds assumption where applicable. No violations were detected.

**Table 7:** Logistic regression models.

Model	OR (IC95%)	*p*	Nagelkerke *R*^2^	Hosmer–Lemeshow *χ*^2^ (gl)	*p* HL
Model 1 (Raw)	2.10	0.011	0.093	3.76 (7)	0.808
Model 2 (adjusted SES)	1.47	0.230	0.101	3.76 (7)	0.808

The raw model showed a Nagelkerke *R*^2^ of 0.093, while the adjusted model showed a Nagelkerke *R*^2^ of 0.101. Hosmer–Lemeshow tests indicated good model calibration in both cases (*p* = 0.808). These results confirm that the models demonstrate adequate goodness-of-fit, although the explanatory power is modest, which is expected in behavioral and social health research. The model explains approximately 10% of the variance.

**Figure 1:**
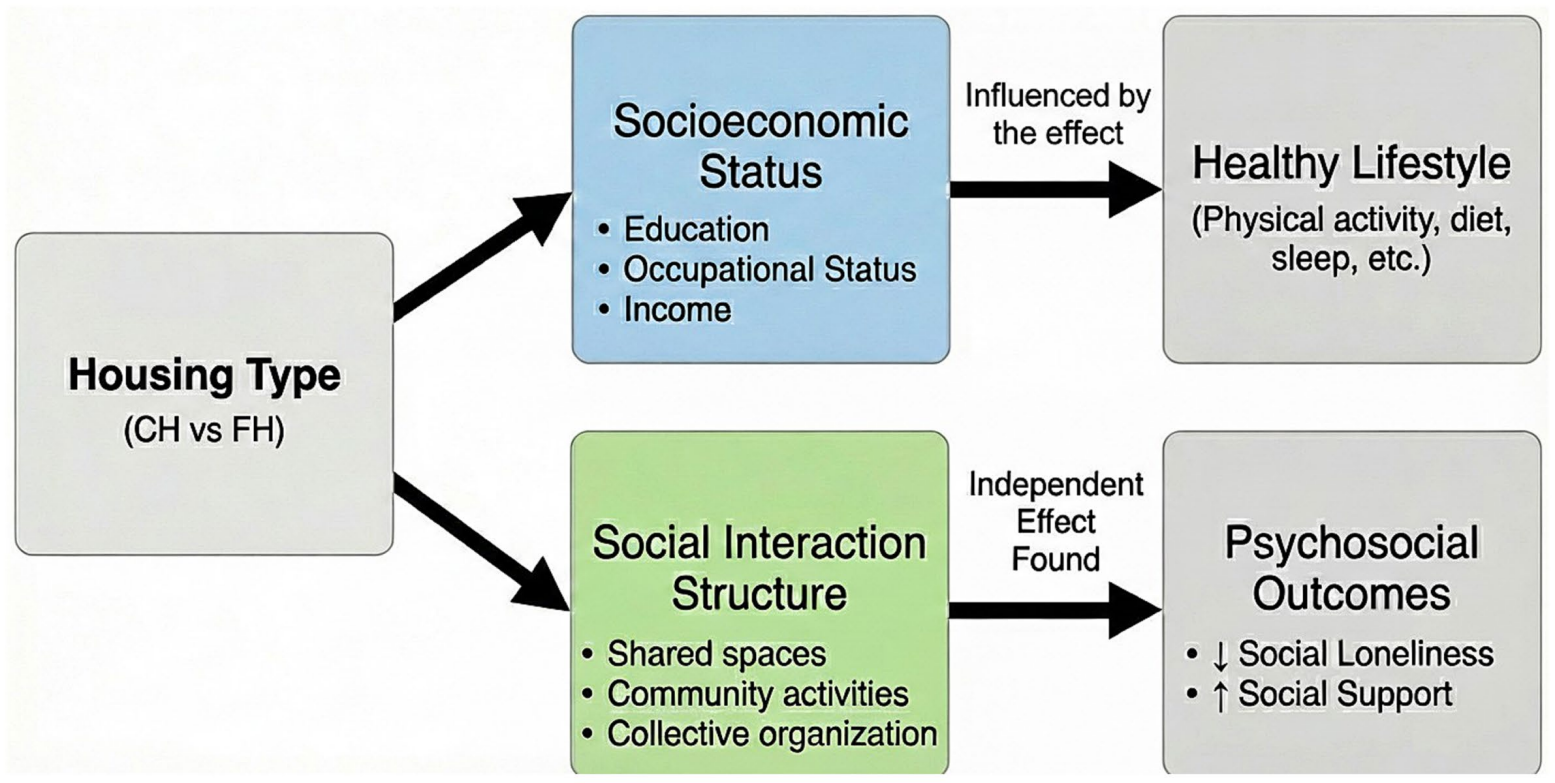
Conceptual model of the relationship between cohousing and health outcomes. Original work.

The inclusion of socioeconomic variables reduces the effect and renders it non-significant. Socioeconomic differences explained the apparent lifestyle advantages observed in cohousing residents. However, cohousing was independently associated with reduced social loneliness and increased social support. These findings suggest that the primary health contribution of cohousing may operate through psychosocial rather than behavioral pathways. Figure 1 outlines the conceptual framework underpinning the study. Socioeconomic factors can confound the association between housing type and lifestyle habits. Conversely, cohousing can exert an independent effect on psychosocial outcomes through increased social interaction and community participation.

## Discussion

The main objective of our study was to analyze the socio-demographic and economic profile, objective and subjective health status, psychosocial wellbeing, and cognitive functioning of older people living in CH compared to those living in FH.

The main findings are listed below:

(a) Older people living in CH have higher levels of education, higher occupations, and moderate to high incomes compared to those living in FH.
(b) Cohousing residents have a healthier lifestyle than those living in FH.
(c) The healthier lifestyle of cohousing residents disappears after adjusting for educational level, occupation, and income. Cohousing acts as an environment that brings together individuals who are already predisposed to healthy lifestyles, rather than being the primary factor that generates them.
(d) Living in CH remains independently associated with lower social loneliness and higher social support after adjusting for educational level, occupation, and income.
(e) Residents in CH have a higher number of illnesses and a poorer self-perception of their health than those living in FH.
(f) Residents in CH experience both positive and negative emotions to a greater extent than those living in FH.
(g) Residents in CH give and receive more social support, have greater social engagement, and stronger social connections than those living in FH.
(h) Older people living in CH perform better on cognitive tests involving memory and executive functions (particularly the executive component of language related to verbal fluency) than people living in FH.

Although the study reveals that socioeconomic status explains much of healthy lifestyle behavior, cohousing maintains an independent association with psychosocial wellbeing. This means that the cohousing environment itself is an engine for flourishing by reducing social loneliness and increasing perceived support, factors that the literature links to a full and healthy life in old age.

This ‘contextual effect’ of the residential environment is what reduces social loneliness and increases social support, regardless of whether the individual has a high level of income or education. Cohousing involves a residential paradigm shift that offers not only a roof over one’s head but an environment designed for self-fulfillment and deep social connection.

### Sociodemographic and economic variables

The first objective was to analyze the socio-demographic and economic differences between older people living in CH and those living in FH.

The group living in CH is characterized by a high level of education; more than half of them have completed higher education and have attained high professional qualifications. Most of them have worked as professionals or company managers, and their average monthly income is higher than that of those in FH.

Our results are consistent with other studies that indicate that CH is a particularly attractive housing alternative for older people with medium-high levels of education and economic resources, since a high initial financial contribution is required, which for many people with lower economic levels can represent a barrier to accessing cohousing (17, 91). Generally, a high initial financial contribution is required, which often acts as a barrier to accessing cohousing.

Higher levels of education are linked to greater personal and intellectual interests (92), major social interests (93), increased empowerment (94, 95), and interest in entrepreneurship (96). In many cases, this is related to the professional activity carried out over the course of life (93, 94, 96). Several studies have indicated that a high level of education is associated with a high interest in developing and maintaining a healthy lifestyle, which in turn is related to having the economic resources that allow access to a greater diversity of social, cultural, and physical activities, as well as to healthier eating (97), and to being able to afford housing in a cohousing community. It is also associated with increased cognitive reserve (98, 99), reduced risk of cognitive decline (100, 101), high levels of PA (102, 103), increased satisfaction with life (104), and a more extensive social network (105, 106).

### Objective and subjective health status

The second objective was to study the differences in health status and lifestyle between older people living in CH and those living in FH.

In our study, multimorbidity was analyzed as the sum of the *number of diseases* of each participant, and statistically significant differences were found between the two groups. People living in CH had a higher average number of diseases than people in FH. Although both values are similar to those reported in other studies such as Pereira de Sousa et al. (107) or Pérez et al. (108), where the average number of diseases is between 2 and 3, or in Gutiérrez-Valencia et al. (109) with an average of 4.3 diseases, we found it noteworthy that people living in CH have more pluripathology than people living in FH. This led us to explore this idea in the scientific literature and to consider whether suffering from an illness is a further reason for older people to choose to live in a friendly and safe environment where they receive support and care to face potential health challenges in the future. We have only seen this aspect reflected in the study of Wardle, which compared people over 50 living in intergenerational cohousing with people living in nearby neighborhoods. It found a higher incidence of disease and disability than in the intergenerational cohousing group. However, this author found that only 16% of those living in cohousing needed care, compared to 33% of people living in the traditional housing area nearby (110). Glass reports that 80% of surveyed participants indicated they would be very likely to ask other residents for help with personal care or household tasks, which was one of the reasons they wanted to live in a community (17). It may also be that only people with high cognitive and economic capacity who have some illness are able to manage such a project while thinking about their own future when they need care.

These results, combined with our direct knowledge of other groups of older adults in CH, lead us to believe that having an illness can influence the decision to seek cohousing as a preferred housing alternative for old age, given that these are environments where healthy aging is promoted and residents care for and support one another.

Scientific literature and study data indicate that higher educational and economic levels are intrinsically associated with a greater interest in maintaining a healthy lifestyle (better diet, greater physical activity, and fewer toxic habits). Therefore, cohousing residents may have better habits not because of the residential environment itself, but because of their cognitive reserve and prior resources. Our results indicate an initial positive association between cohousing and a healthy lifestyle (regression model 1). However, when adjusted for education, occupation, and income (regression model 2), the difference between groups in the healthy living index disappears and is no longer statistically significant. This demonstrates that it is socioeconomic factors, rather than the type of housing, that explain the lifestyle advantage.

In our view, there is a pressing need for further studies quantifying the impact of lifestyle-related factors on general health, life expectancy, and health and social expenditures among older people living in CH, similar to the study conducted by Lo et al. on the general population (111). That study found that individuals who adhered to five of the healthy lifestyle habits experienced a gain of 7.13 years in life expectancy (95% CI: 1.33–11.11; *p* = 0.02) and a 28.12% reduction in annual health expenditure (95% CI: 4.43–57.61%; *p* = 0.02), compared to those who followed only one or none of the habits.

It would also be valuable to follow the line of research by Reyes et al. to understand the effect of moving to a nursing home on the physical health and emotional state of older adults, as well as to study whether their expectations have been met, and the gains and losses of moving to a nursing home. Reyes et al. analyzed the impact of moving to a nursing home on perceived health, mental health, and social support in middle-aged people. Their results indicated improvements in perceived health and mental wellbeing, especially among men, although these effects were not statistically significant (112). Further research in this area is highly desirable.

In relation to toxic habits, the high alcohol consumption of FH residents (72.7%) compared to CH residents (27.7%) is striking. Our results for the group of CH residents are lower than those described in the scientific literature, while in FH they are much higher. In the recent study by Castro et al., the authors estimate that 30–40% of people over 65 years of age consume alcohol on a regular basis, with around 10% engaging in high-risk drinking (113). The alcohol consumption in FH may be related to less daily social contact, boredom, or apathy, which can induce consumption to try to alleviate discomfort. In contrast, living in a CH facilitates social contact, promotes participation in activities inside and outside the community center, and fosters a sense of community and the importance of self-care. Thus, in many community living groups, residents have agreed not to smoke or consume alcohol to avoid risks that would have to be borne or dealt with by other members of the community.

With respect to PA, people in CH maintain a more active life than people living in FH. In the CH group, the percentage of people who perform PA is much higher than in the studies reviewed, so we believe that living in a community seems to favor the performance of PA. Residents in CH show a greater motivation to engage in activities, cooperation and reciprocity, self-care, and the ability to energize and lead some cohabitants who mobilize others to carry out activities, with the benefits it implies for health and wellbeing (114).

In future studies, it is of interest to analyze PA by age ranges and determine whether there is a decreasing trend with age in people living in CH, as found in the study by McGuire et al., where PA decreased from 23.0% in the 65–69 age group to 13.6% in the over 85 age group (115), or in the study by Mummery et al. (116), where the difference between the 60–64 age group and the over 80 age group was 24.2% (115). Alternatively, it could be examined whether there is an increase in PA with increasing age, as in the study of Sims et al., where the 70–74 age group had higher PA than the 64–69 age group (90).

It is also interesting, as in other aspects, to study longitudinally the people who live in CH and analyze whether the practice of PA is reflected in a better state of health and a better quality of life. The scientific evidence clearly shows that being PA and having a healthy diet, together with not smoking and not consuming alcohol, are fundamental for the maintenance of health and wellbeing in older people (117, 118).

We assessed functional ability through the application of ADL scales, BADL, and IADL. Our results indicate that the vast majority of participants are independent. However, regarding IADLs, residents in CH scored lower on the Lawton Index. We believe this is due to the fact that the item “cooking meals” does not accurately reflect the actual functionality of CH residents, as they typically benefit from a daily meal service provided in the shared common facilities of the cohousing arrangement. This highlights the need to adapt IADL assessment tools to the characteristics of the population under study, a situation also observed in institutional settings such as nursing homes (119).

Moreover, residents in CH engage in other types of instrumental activities that are not captured by standard assessments but are highly indicative of functional capacity. These include responsibilities related to governance, household management, coordination, supervision, and implementation of shared services, as well as maintenance of common areas and programming of community activities. The ongoing involvement of residents in the management and sustainability of CH requires a high degree of independence.

As for self-perception of health, FH participants reported a more positive perception of their health. At first glance, these findings may seem contradictory, especially considering the healthier lifestyle observed among CH residents and the well-established link between such lifestyles and better self-rated health (120, 121). However, consistent with previous research, we believe these results may be explained by the dynamics of communal living. Older adults living with peers may become more aware of their own health limitations by observing and comparing themselves to others, potentially leading to a more pessimistic or negative self-assessment of their own health (122). Conversely, older adults living in FH may underreport health issues, either due to a genuine sense of independence or, as suggested in the literature, out of fear of acknowledging the need for family support, professional care, or a potential move to home care (123–125).

### Psychosocial profile

The third objective of this study was to examine whether there are differential characteristics in the psychosocial status of older adults living in CH compared to those living in FH.

First, a positive attitude toward one’s own aging has, according to scientific literature, a positive impact on wellbeing. In our study, when applying the assessment instrument used, no significant differences were found in total scores between the two groups. However, the individualized analysis of items 1, 4, and 5 suggested that CH residents have a more positive perception of aging than those in FH. Perhaps this more positive attitude toward the aging process itself influences self-care, the promotion of physical activity, involvement in social and self-management activities typical of CH, and this, in turn, results in the wellbeing of the people.

Levy and Myers found that older adults with a positive attitude toward aging experienced better health outcomes, increased longevity, greater self-care, and a stronger will to live (126). In another study, Levy et al., using longitudinal data from the Ohio Longitudinal Study of Aging and Retirement (OLSAR), reported that individuals with more positive self-perceptions of aging lived 7.5 years longer than those with more negative perceptions, noting that this effect was partially mediated by greater vital optimism. According to the authors, this effect was also partially mediated by higher optimism for life (127).

Authors such as Lamont et al. and Siebert et al. point out that it is negative stereotypes and not facts that cause underperformance on cognitive and physical tasks (128, 129). Our results seem to follow this same line and support negative self-perceptions of aging as a risk factor in old age.

In future studies, we consider it relevant to analyze the influence of self-perception of aging through longitudinal follow-up of participants to examine its relationship with factors such as longevity, objective and subjective health, and social support.

Regarding the affects, participants living in CH reported both higher positive and higher negative affect than those living in FH. Living in CH involves numerous shared experiences as well as conflict management and resolution, which tend to be more intensive and varied than those experienced in FH, due to the difficulty of reaching a consensus among all cohabitants. Residents in CH assume full responsibility for managing services and shared spaces. While this responsibility is often viewed as rewarding, it is also described as exhausting (130). In the Dual Affect Model, we find a theoretical framework of the psychology of emotions that posits that affective experience is not a single dimension ranging from ‘bad’ to ‘good,’ but rather is composed of two independent dimensions, not two opposite poles: positive affect (level of energy, enthusiasm, and pleasurable engagement; High: energy, enthusiasm, pleasure; Low: sadness, lethargy, lack of energy) and negative affect (subjective discomfort and unpleasant engagement; High: discomfort, anger, guilt, fear, nervousness; Low: calm, serenity, tranquility). A person can score high on positive affect and also high on negative affect at the same time (e.g., being excited but nervous about a new challenge), or low on both (state of tranquility and lack of energy) (131, 132).

The higher level of negative affect recorded among residents is justified as an intrinsic result of the social intensity and self-management responsibilities of the model. We believe that the specific mechanisms explaining this phenomenon are:

- Self-management exhaustion: CH residents assume full responsibility for managing common services and spaces. Although this work is perceived as rewarding, it is also described as ‘exhausting’ (131). This type of exhaustion has a stronger impact on increasing negative emotions than on decreasing positive ones (132).
- Complexity of consensus. Unlike life in an FH, the CH requires consensus among all cohabitants for decision-making. This process of collective negotiation is difficult, generating interpersonal tensions and conflicts that increase negative affect (130).
- Deep emotional bonds. Community living involves sharing intense life experiences. This exposes residents to emotionally demanding situations, such as mourning the death of companions or the frustration of receiving less social support than expected at specific times (131, 132).
- The ‘*paradox of intensity’*. The CH functions as an ecosystem that amplifies both emotional poles. Daily contact and active participation foster a high level of positive affection (due to the sense of belonging and support), but this same relational density inevitably leads to a higher frequency of friction and emotional challenges.

Negative affection should not be interpreted as a sign of failure of the model, but rather as the ‘emotional cost’ of a participatory and self-managed life, where the complexity of human relationships and the governance of the center demand constant psychological effort.

Living in a community with a shared lifestyle may increase both positive and negative affect, depending on factors such as the quality of social relationships and the experiences within the communal environment. Positive affect may be enhanced through social interaction, participation in shared activities, emotional support, and the sense of belonging fostered by the community. At the same time, negative affect can also be influenced by communal living. Interpersonal conflicts, feelings of emotional loneliness, receiving less social support than desired, or experiencing grief following the death of fellow residents are factors that may contribute to increased negative affect. In summary, communal living is a source of both positive and negative emotions, with the quality of social relationships and individual coping capacity playing a crucial role in shaping the emotional experience within the cohousing environment.

With respect to feelings of loneliness, the results indicate the absence of statistically significant differences in the total score on the DJGLS-6 scale. Both groups score on average under 2, which may be interpreted within the range of not feeling lonely.

However, we analyzed each item of the scale individually and observed statistically significant differences in precisely two of the items related to social or relational loneliness (items 2 and 4). The results indicate that the CH group experiences less relational loneliness than the FH group. The social network of people living in FH is likely to be smaller; daily social contact with friends in FH is probably less frequent and involves fewer people than that of residents in communities or CH, who interact daily with their peers in various activities inside and outside the CH.

Nonetheless, as we have noted, there are no differences in the total score on the loneliness scale, only in the two items related to social loneliness. In general, we believe that the absence of feelings of loneliness in both groups may be related to various factors, such as the participants’ relatively young age, good emotional state, a positive self-concept regarding their aging, a zest for life, and life satisfaction.

In general, the study participants are “young” older adults (*M* = 73.74 years). Numerous studies suggest that older adults face a greater risk of loneliness as they age, particularly from the age of 80 onwards; therefore, perhaps age is a factor to consider in the results. Pinquart and Sörensen found a weak positive correlation between loneliness and age, which became greater as one grew older (133). In Western countries, the prevalence of loneliness among people over 65 years ranges from 24–40%, increasing substantially with age, with approximately 50% of individuals aged 80 and older reporting frequent feelings of loneliness (134–137). Older adults are generally more vulnerable to loneliness and social isolation than other age groups (138–140); therefore, once again, we believe that being able to conduct longitudinal studies with older adults who are aging in CH communities is a line of research that needs to be explored also in the field of unwanted loneliness.

Regarding social support, significant differences were observed between the two groups. Participants living in CH indicated feeling significantly higher levels of social support than those who lived in FH. Our findings are consistent with existing scientific literature indicating that CH offers older adults increased opportunities for social contact and support (30, 39, 141). Marke et al. studied social support behaviors in two groups of older adults: those living in CH and those in FH. Their findings revealed that CH residents both gave and received significantly more social support than those living independently. Moreover, mutual support among CH residents increased over time, fostering a stronger sense of community (142).

In general, studies have demonstrated that social interactions in CH reduce loneliness among older adults (143, 144), provide opportunities for social and emotional support, companionship, and mutual benefits for all residents (39), foster wellbeing (145), benefit physical and mental health, and positively impact residents’ quality of life (53, 146).

In our study, the socioeconomic status of participants in CH confounds lifestyle (behavioral) outcomes but not psychosocial outcomes. Our results indicate that CH continues to be associated with less social isolation and greater social support, independently of socioeconomic status. Multivariate regression models (Model 2) show that, after adjusting for educational level, occupation, and income, CH continues to be independently associated with greater perceived social support (*p* = 0.006) and less social loneliness (*p* = 0.013). This indicates that the protective effect comes from the ‘social ecosystem’ and not from the residents’ status.

The shared lives have allowed them to develop a sense of community, understood as a feeling of belonging and connection among members, and the perception that needs will be met through the commitment to being together (147). The sense of community is closely related to the community participation of CH residents. Research suggests that social interrelations are associated with better mood, contribute to improved physical and mental health, greater social engagement, and enhance psychological wellbeing (148, 149).

Regarding social engagement, the differences between groups in our results were highly significant. CH participants have a long and extensive history of social commitment (including social, civic, recreational, cultural activities, volunteering, and associational involvement) compared to the FH group.

Our results indicate that people living in CH have had greater social engagement throughout their lives, which they continue to maintain in the CH setting. Several authors have studied social engagement among older adults living in CH. Nguyen and Levasseur analyzed the impact of cohousing on older adults’ social engagement and emphasized the importance of situating cohousing developments in environments that promote physical and social interaction among residents, as well as openness to sharing resources with other local communities to create a supportive environment conducive to active and healthy aging (150). Puplampu described the experiences of older adults forming and living in a CH community and its impact on aging. Findings showed that living in CH promoted social engagement, social interaction, security, and mental health among older adults, while also providing support services (151).

Compared to traditional FH, this model fosters a greater sense of community, reduces loneliness and isolation, strengthens social support networks, and promotes greater psychological wellbeing (152, 153). Beyond the physical dimension of the living space, CH demonstrates that the way we live together and organize ourselves collectively can become a key factor for a healthier and more fulfilling life (154–156).

Psychosocial benefits are an intrinsic added value of the residential model, making it a particularly powerful social determinant of health for public policies, since its effectiveness does not depend on the prior wealth of individuals, but on the way in which they decide to organize themselves and live together.

### Cognitive profile

The fourth objective focused on analyzing whether older adults living in CH demonstrate better cognitive performance compared to those living in FH. Regarding general cognitive status, no significant differences were found; both groups showed normal performance with mean scores above 28 points. We used additional assessments for verbal episodic memory, semantic memory, executive function, working memory, and attention.

In our results, statistically significant differences were found between groups in the three tasks that assess verbal memory: the number of items recalled in the first and fourth trials, and delayed recall. Significant differences were observed, with higher average scores in the CH group compared to the FH group. Similarly, semantic memory and executive language function, evaluated with phonological and semantic fluency tasks, showed statistically significant differences.

The cognitive advantage observed in CH residents, especially in domains such as verbal episodic memory and semantic fluency, can be explained by several interconnected psychological and psychosocial mechanisms that act as powerful brain stimulants (157, 158). These mechanisms include, first and foremost, the high cognitive demands of self-management. CH requires residents to take on collective governance of the project. Organizing community life, defining rules, allocating budgets, and coordinating support services requires constant exercise of executive functions and decision-making, which act as a continuous stimulus for mental capacity. The second mechanism is related to “active reciprocity.” CH transforms passive coexistence into a system of mutual support and active participation that gives meaning to life and maintains cognitive capacity (159). The third factor is related to social intensity and complexity. Social relationships in CH are more frequent and varied than in FH, which increases language use and memory (160). In addition, the psychological effort required to manage conflicts and reach consensus demands high cognitive performance. The fourth factor relates to the enhancement of cognitive reserve. In addition to having high levels of education and professional qualifications, the CH environment can act as a catalyst that allows residents to continue cultivating intellectual and social interests, better protecting them against cognitive decline. The fifth mechanism is the safety net and social support network provided by living in a nursing home. A stimulating social ecosystem forces older adults to remain mentally active and emotionally engaged, resulting in superior neuropsychological performance compared to those living at home.

Before concluding, we would like to highlight the importance of “*active reciprocity”* as a central concept in the senior cohousing model, which transforms passive coexistence into a dynamic ecosystem of mutual support that protects against cognitive decline, reduces relational loneliness, and improves quality of life through shared social engagement.

We believe that the role of *“active reciprocity”* in health manifests itself in the following dimensions:

1 Stimulation of cognitive ability.

*Active reciprocity* forces residents to abandon passive roles and become organizers, managers, or mediators within their community. Self-management and the organization of community life place high cognitive demands that act as a stimulant for mental capacity. This constant exercise in collective decision-making and problem-solving is linked to better performance in critical areas such as verbal episodic memory and semantic fluency.

2 Psychosocial wellbeing and flourishing.

The exchange of support (both giving and receiving) is the basis of the concept of flourishing or personal growth in old age. Taking on responsibilities within the group helps maintain a sense of purpose and usefulness, contributing to a full and meaningful life. Reciprocity fosters a robust social connection that specifically reduces relational loneliness (the feeling of not having someone available in case of need), an effect that remains even when controlling for individuals’ socioeconomic status.

3 Physical health and lifestyles.

Reciprocity acts as a motivating force for maintaining healthy behaviors. Mutual support and cooperation within the community facilitate group physical activity. The ability of some residents to ‘energize’ and mobilize others acts as an incentive that improves adherence to exercise. The environment of mutual care fosters shared responsibility for one’s own health and that of others, resulting in more active and healthy lifestyles compared to those who live independently.

4 The ‘*paradox*’ of negative affection.

An important finding from the sources is that this *active reciprocity* and the social intensity it entails come at an ‘emotional cost.’ Residents report higher levels of negative affect due to the responsibility of managing services, the difficulty of reaching consensus, and the handling of interpersonal conflicts. However, this exhaustion is interpreted as a sign of an emotionally intense and engaged life, as opposed to the apathy that can arise in other housing models.

This study represents a significant advance in the scientific literature on senior cohousing. Its main strength lies in its originality within the Spanish context. To date, there have been no studies in Spain that quantitatively and comparatively analyze the sociodemographic profile, health status, psychosocial wellbeing, and cognitive status of older people in CH versus FH. One outstanding aspect is the sample size (*n* = 171), which is larger than that of many previous articles on senior cohousing, which tend to be qualitative or based on much smaller samples. The study is not limited to superficial surveys but employs a multidimensional assessment and uses standardized tools.

The most relevant finding is that cohousing has an impact on loneliness and social support regardless of a person’s socioeconomic status. While maintaining a healthy lifestyle (diet, exercise) usually requires a higher socioeconomic status (education, occupation, income), social support in cohousing is a ‘golden point’ of the model because it is a purely relational benefit. The study shows that socioeconomic status is not a significant predictor of social support in the adjusted models (*p* > 0.29 in all cases), reinforcing the idea that money does not buy the quality of the social fabric in this community.

The study offers data-driven guidance for public policymakers and health strategists.

Despite the strengths and findings of this study, several limitations should be acknowledged. First, the fact that all CH participants belonged to a single community may limit the generalizability of the results and external validity, as conditions could differ in other cohousing groups. Second, our comparison group consists of older adults living in HF. A possible representativeness bias could arise from this group, as the participants represent a particularly active segment of the population, with a socioeconomic status that may not reflect the full diversity of the older population in Spain. Given that the study compares two groups with high levels of social engagement and specific housing resources, the results should be interpreted with caution, as they may underestimate the differences that would be found when comparing cohousing with other types of housing. Third, with the exception of neuropsychological assessments, all variables were self-reported, introducing the possibility of social desirability bias, especially in the CH group, who may want to “validate” their lifestyle. Longitudinal research is needed to explore how lifestyle, life satisfaction, self-perception of aging, social support, and other psychosocial determinants interact over time in health and longevity.

## Conclusion

The main finding of this study is that socioeconomic differences largely explained the association between cohousing and a healthy lifestyle. However, cohousing remained independently associated with lower social loneliness and higher perceived social support.

The community environment fosters closer social bonds and connections among those living together, promotes independence, and enhances overall wellbeing during old age.

Senior cohousing emerges as a transformative housing alternative that transcends physical space to become a social ecosystem of mutual support that protects the mental and physical health of older adults. The findings suggest that self-management and active participation in the design of community life are decisive factors for healthy aging, fostering not only functional autonomy but also emotional wellbeing and social connection.

## Data Availability

The raw data supporting the conclusions of this article will be made available by the authors, without undue reservation.

## Footnotes

1
https://www.who.int/publications/i/item/9789240015128
